# LINC00261: a burgeoning long noncoding RNA related to cancer

**DOI:** 10.1186/s12935-021-01988-8

**Published:** 2021-05-22

**Authors:** Menggang Zhang, Fang Gao, Xiao Yu, Qiyao Zhang, Zongzong Sun, Yuting He, Wenzhi Guo

**Affiliations:** 1 Department of Hepatobiliary and Pancreatic Surgery, The First Affiliated Hospital of Zhengzhou University, No. 1 Jianshedong Road, Erqi District, Zhengzhou, 450052 China; 2 Key Laboratory of Hepatobiliary and Pancreatic Surgery and Digestive Organ Transplantation of Henan Province, The First Affiliated Hospital of Zhengzhou University, Zhengzhou, 450052 China; 3 Open and Key Laboratory of Hepatobiliary & Pancreatic Surgery and Digestive Organ, Transplantation at Henan Universities, 450052 Zhengzhou, China; 4 Henan Key Laboratory of Digestive Organ Transplantation, Zhengzhou, 450052 China; 5 Health Management Center, Binzhou People’s Hospital, Binzhou, 256600 China; 6 Department of Obstetrics and Gynaecology, The Third Affiliated Hospital of Zhengzhou University, Zhengzhou, 450052 China

**Keywords:** Long noncoding RNA 261, Biomarker, Human cancers, Biological function, Therapeutic target

## Abstract

Long noncoding RNAs (lncRNAs), are transcripts longer than 200 nucleotides that are considered to be vital regulators of many cellular processes, particularly in tumorigenesis and cancer progression. long intergenic non-protein coding RNA 261 (LINC00261), a recently discovered lncRNA, is abnormally expressed in a variety of human malignancies, including pancreatic cancer, gastric cancer, colorectal cancer, lung cancer, hepatocellular carcinoma, breast cancer, laryngeal carcinoma, endometrial carcinoma, esophageal cancer, prostate cancer, choriocarcinoma, and cholangiocarcinoma. LINC00261 mainly functions as a tumor suppressor that regulates a variety of biological processes in the above-mentioned cancers, such as cell proliferation, apoptosis, motility, chemoresistance, and tumorigenesis. In addition, the up-regulation of LINC00261 is closely correlated with both favorable prognoses and many clinical characteristics. In the present review, we summarize recent research documenting the expression and biological mechanisms of LINC00261 in tumor development. These findings suggest that LINC00261, as a tumor suppressor, has bright prospects both as a biomarker and a therapeutic target.

## Background

It is well-known that cancer is the leading cause of death worldwide [1–3]. Although many clinical therapies exist, the death rate for cancer remains high [4, 5] and prognoses remain poor due to a lack of effective biological markers for early diagnosis. Therefore, the identification of novel biomarkers is very important for improved diagnosis and treatment [6, 7], and lncRNAs have recently been shown to be a group of such novel biomarkers [8–10].

Noncoding RNAs (ncRNAs), are functional RNAs which don’t encode proteins, including microRNAs (miRNAs), circular RNAs (circRNAs), and lncRNAs functions. Most ncRNAs operate as RNA-protein complexes and exert many cellular functions, including miRNAs and lncRNAs [11]. ncRNAs regulate specific gene expression at the transcriptional level, through regulating transcriptional, post-transcriptional, and post-translational processes [12]. As a result, through their profound effect on protein expression and function, ncRNAs have participated in many cellular events such as proliferation, migration, invasion, differentiation, apoptosis, immune responses, etc. [12]. Considered important regulators of tumor progression, lncRNAs are noncoding RNAs of more than 200 nucleotides [13], that are transcribed by RNA polymerases II and III without the ability to code for proteins [14, 15]. Accumulating evidence indicates that many lncRNAs are abnormally expressed in many tumor types [16–18], and play crucial roles in a variety of cellular events, including the regulation of gene transcription [19, 20], protein translation [18, 21], post-transcriptional modification [22, 23], and messenger RNA (mRNA) processing [24, 25]. Most usually, lncRNAs mainly sponge miRNAs and decrease the level of special miRNAs, which accelerate the degradation of mRNAs. In addition, lncRNA expression has been related to the occurrence [26, 27], progression [28, 29], metastases [30, 31], and prognoses of a variety of tumors [32, 33].

The transcription site for LINC00261 is located on the 20th chromosome from site 22,560,552 to 22,578,642 [24, 34]. LINC00261 has been widely reported to be a tumor inhibitor in a variety of cancers, involving in many cellular processes [35, 36]. For example, one study found that LINC00261 was a potential biomarker for endometrial carcinoma prognosis [37]. LINC00261 inhibits tumor-cell growth primarily by inhibiting cell proliferation and promoting cell apoptosis [34, 36]. Additionally, LINC00261 has been shown to reduce tumor-cell invasiveness by inhibiting the epithelial-mesenchymal transition (EMT) [38, 39]. LINC00261 expression has also been reported to reduce the proliferation and migration of breast cancer cells [40]. These studies mainly focused on the anti-tumor functions of LINC00261 and its different roles in the pathogenesis of different tumor types.

The present review has two main objectives. The first is to survey of the most recent LINC00261 studies related to cancer, enlarging the map of its influence and highlighting new research insights. The second is to discuss how these recent studies have guided innovation. It will be greatly helpful for researchers to learn about the general research status.

### The upstream mechanism underlying the dysregulation of LINC00261

Many studies have revealed that lncRNAs can be regulated in both gene level and transcription level. At the genome level, both the increased copy number of special oncogene and the chromosome deletion can influence lncRNA level [41, 42]. Additionally, lncRNAs also can be regulated by transcription factor [43], histone modification [44], and the methylation level of promoter region [45]. The lncRNAs stability even can be mediated through post-transcriptional mechanism [46]. In terms of LINC00261, Liu et al. [47] demonstrated that LINC00261, as a tumor suppressor, can be inactivated by methylation of promoter region, while the demethylation could upregulate LINC00261 and inhibit the progress of pancreatic cancer. Therefore, in pan-carcinoma, we summarize the expression and clinical features of LINC00261 in the following.

### The expression and clinical characters of LINC00261 in multiple human cancers

An increasing number of studies have indicated that LINC00261 plays vital roles in a variety of cancers, as discussed below. This overview of the many types of cancer related to LINC00261 expression expands the map of LINC00261 and influences further researches. The expression of LINC00261 and its clinical-characteristic associations in multiple tumors are shown in Table 1.

**Table 1:** The expression of LINC00261and its clinical character in multiple cancers

Cancer type	Property	Expression	Cases	Clinical character	Prognosis	PMID
Pancreatic cancer	Suppressor	⬇	229	Prognosis	Favorable	30,210,701
		⬇	/	Prognosis	Favorable	32,414,223
		⬇	40	Prognosis	Favorable	32,929,371
		⬇	/	Prognosis	Favorable	33,122,827
Gastric cancer	Suppressor	⬇	/	Prognosis	Favorable	27,439,973
		⬇	/	Prognosis	Favorable	27,878,953
Colon cancer	Suppressor	⬇	138	Prognosis	Favorable	31,850,713
Lung cancer	Suppressor	⬇	150	Prognosis	Favorable	29,272,004
		⬇	52	Prognosis	Favorable	31,190,356
		⬇	78	Prognosis	Favorable	32,607,060
		⬇	881	Prognosis	Favorable	32,181,394
Hepatocellular carcinoma	Suppressor	⬇	45	Prognosis	Favorable	29,278,875
		⬇	317	Prognosis	Favorable	29,761,859
		⬇	44	Prognosis	Favorable	33,520,374
		⬇	74	Prognosis	Favorable	30,377,132
Breast cancer stem cells	Suppressor	⬇	103	Prognosis	Favorable	33,274,565
Laryngeal carcinoma	Suppressor	⬇	66	Prognosis	Favorable	29,774,690
Prostate cancer	Suppressor	⬇	83	Prognosis	Favorable	33,013,201
Choriocarcinoma	Suppressor	⬇	60	Prognosis	Favorable	27,983,929
Cholangiocarcinoma	Oncogene	⬆	50	Prognosis	Poor	31,812,439

### Pancreatic cancer

For cancer-related death rankings, pancreatic cancer (PC) is number seven worldwide [48]. With surgery being its most effective treatment, however, PC prognoses are still extremely poor [49–51]. Müller et al. [52] reported that LINC00261 expression was significantly down-regulated in PC, and Dorn et al. [53] also indicated that LINC00261 expression was significantly reduced in the squamous subtype. This last report, using bioinformatics, also showed that LINC00261 expression was negatively correlated with both tumor grade and tumor stage, and significantly related to positive disease outcomes [53]. The up-regulation of LINC00261 has been shown to obviously suppress PC cell proliferation and migration both in vitro and in vivo [47]. In addition, Zhang et al. [54] reported that LINC00261 expression was down-regulated in both PC tissues and in serum, with the level of expression being negatively correlated with clinical stages. LINC00261 expression has also been reported to suppress PC glycolysis and proliferation and to induce both cell-cycle arrest and apoptosis [35]. This study further our understanding of PC pathogenesis and suggests that LINC00261 may be a PC prognostic and therapeutic target [35].

### Gastric cancer

With the third highest cancer-related death ranking, the gastric cancer (GC) mortality rate is 75 % [55], and it represents the fourth most-common tumor type in the world [56]. Due to imperceptible symptoms in the early period of GC, the rate of early diagnosis remains low [57–59]. Therefore, further studies of effective GC targets are crucial for improving both early diagnosis and the prognoses of GC patients. Compared to expressions in normal gastric tissue and para cancerous tissue, LINC00261 expression was found to be downregulated in GC [60, 61], and low LINC00261 expression was correlated with tumor stage, lymphatic metastases, and tumor invasiveness [61]. In addition, low LINC00261 expression was reported to predict poor prognosis and active cell motility, resulting in the suppression of tumor metastasis both *in vitro* and *in vivo* [61]. Moreover, up-regulated LINC00261 expression was reported to suppress cell motility by inhibiting the EMT [62]. These results suggest that LINC00261 can be regarded as a potential target for GC to prevent metastasis and invasiveness.

### Colorectal cancer

Colorectal cancer (CRC) is one of the deadliest types of digestive system tumors, caused by interactions between genetic and environmental factors [63–65]. Interestingly, LINC00261 expression was reported to be low in both colon-cancer cell lines and tissues and in cisplatin-resistant cells [63]. In addition, LINC00261 over-expression was found to promote cell apoptosis and to inhibit cell viability, migration, invasiveness, and proliferation [63, 66]. Moreover, the up-regulation of LINC00261 was found to suppress cell-colony formation and to accelerate apoptosis [66]. Clinically, the low expression of LINC00261 was reported to be an independent risk factor for CRC patients that influenced recurrence-free survival time after surgery operation [67]. These findings suggest that LINC00261 may also be a potential therapeutic target for CRC.

### Lung cancer

The leading cause of cancer deaths globally is lung cancer (LC) [68–70], with 80–85 % of cases being non-small-cell lung cancer (NSCLC) [71]. Liu et al. [72] found that, compared to adjacent normal lung tissues, LINC00261 expression was down-regulated in NSCLC tissues and that its expression was correlated with TNM stage, lymph-node status, distant metastases, and poor overall survival. It was also found to inhibit cell proliferation and metastasis by downregulating Snail expression via the EMT [73]. Furthermore, Shi et al. [34] showed that up-regulated LINC00261 expression in NSCLC cell lines suppressed both cell proliferation and invasiveness, while at the same time accelerating apoptosis. Moreover, LINC00261 was reported to be the most common down-regulated lncRNA in tumor metastasis, and low LINC00261 expression was correlated with poor overall patient survival using five independent lung-adenocarcinoma cohorts (n = 881) [74]. In addition to this, low LINC00261 expression was also found to be an independent indicator for poor NSCLC prognoses [72]. These results further our understanding of NSCLC pathogenesis and indicate another potential target to combat this deadly disease.

### Hepatocellular carcinoma

As the sixth most common cancer worldwide, hepatocellular carcinoma (HCC) represents one of the top causes for cancer deaths worldwide, with more than 700,000 cases diagnosed annually [75–77]. HCC tumorigenesis and progression is closely associated with lncRNAs [78]. Zhang et al. [79] demonstrated that LINC00261 expression was significantly lower in in HCC tissues compared to adjacent noncancerous tissues, and that its expression was significantly correlated with TNM stage, tumor size, and overall HCC patient survival time. In addition, up-regulated LINC00261 expression in HCC cells was shown to suppress cell proliferation, colony formation, invasiveness, and the EMT *in vitro*. For HCC clinical features, Sun et al. [80] showed that LINC00261 was an independent prognostic marker and that HCC patients with lower LINC00261 expression had significantly shortened survival times for both tumor-free survival and postoperative recurrence-free survival. Using HCC-derived cell lines, low LINC00261 expression was reported to significantly promote cell motility [81]. Chen et al. [82] also reported that LINC00261 over-expression inhibited the EMT in liver cancer cells, thereby suppressing migration, invasiveness, and the formation of lung metastatic lesions. Therefore, based on these analyses, LINC00261 expression may serve as prognostic marker for HCC patients.

### Breast cancer

Most prevalent in middle-aged women, breast cancer (BC) is ranked second among common causes for cancer-related deaths in the US [83–85]. At present, lncRNAs identified as being associated with BC include ATB and CCAT1. LINC00261 has also been shown to be downregulated in BC tissues compared to adjacent normal tissues. Up-regulation of LINC00261 has been reported to inhibit both BC-cell proliferation and migration, and the low expression of LINC00261 was reported to be adequate to promote BC tumorigenesis [39]. In addition, Li et al. [40] reported that LINC00261 over-expression inhibited both the viability and motility of BC cells, with potential implications for treatments, so LINC00261 may also be a new potential target for BC.

### Other cancers

For laryngeal carcinoma tissue, the expression of LINC00261 was found to be significantly lower compared to normal tissues, and its expression in the lymph-node-negative metastasis group was significantly higher compared to that of lymph-node-positive metastasis group [86]. LINC00261 was also found to be downregulated and to suppress both cell proliferation and motility in endometrial carcinoma [37]. Similarly, for esophageal cancer, the anti-tumor influence of LINC00261 was determined using 5-fluorouracil (5-FU) to detect increased drug sensitivity in human esophageal cancer cells [87]. Other studies have also demonstrated an inhibitory effect of LINC00261 expression in prostate cancer, choriocarcinoma, and cholangiocarcinoma [38, 88, 89].

### Multiple biological functions of LINC00261 in cancer

LncRNAs generally exert their functions through complex molecular mechanisms, such as the sponging of miRNAs in cancer cells [3, 90, 91]. In addition to discussing LINC00261 expression and its clinicopathological features for the cancers above, the biological functions and molecular mechanisms of LINC00261 are summarized below (see Table 2).

**Table 2:** The biological functions and molecular mechanisms of LINC00261

Cancer type	Property	Functional role(validated)	Related genes/proteins/pathways	PMID
Pancreatic cancer	Suppressor	EMT, motility, invasiveness	FOXA2, Wnt pathway	32,414,223
		Metastasis	Wnt, miR5525p/FOXO3	32,020,223
		Proliferation, metastasis	c-Myc, MYC, E2F	32,929,371
		Progression	FOXA2, cAMP and MAPK	32,590,069
		Cell-cycle arrest, proliferation	IGF2BP1, HIPK2/ERK	33,122,827
Gastric cancer	Suppressor	EMT	Focal adhesion	27,439,973
		EMT	Ubiquitin-proteasome	27,878,953
Colon cancer	Suppressor	Drug resistant, apoptosis, viability	Wnt/β-catenin	29,267,503
		Progression	miR-324‐3p, Wnt/β‐catenin	31,183,860
Lung cancer	Suppressor	EMT	/	31,115,010
		Cell proliferation, invasion, and apoptosis	miR-522‐3p, Wnt	31,190,356
		Metastasis and proliferation	FHL1	31,772,674
		Tumorigenesis, and progression	FOXA2, ERK pathway	30,597,925
		Proliferation and metastasis	Wnt/β-catenin, miR-1269a/FOXO1	32,607,060
		Migration, and proliferation	FOXA2	30,796,052
Hepatocellular carcinoma	Suppressor	Proliferation, invasion, EMT	Notch signaling pathway	29,278,875
		/	PPAR signaling pathway	29,761,859
		Metastasis, migration, invasion, and EMT	FOXA2, TGF-β signaling	33,520,374
		Migration, and invasion	/	30,377,132
Breast cancer	Suppressor	Cell viability, microsphere formation ability, migration, and invasion	/	33,274,565
		Proliferation, migration, and tumorigenesis	NME1-EMT pathway	32,440,206
Laryngeal carcinoma	Suppressor	/	/	29,774,690
Endometrial carcinoma	Suppressor	Proliferation, migration, and invasion	/	30,019,459
Esophageal cancer	Suppressor	Chemotherapeutic response	Metabolic pathway	30,226,808
Prostate cancer	Suppressor	Proliferation, angiogenesis	p38 MAPK, Wnt/β-catenin	33,013,201
Choriocarcinoma	Suppressor	Proliferation, EMT, and cell apoptosis	/	27,983,929
Cholangiocarcinoma	Oncogene	Metastasis, and EMT	/	31,812,439

### Cell proliferation

**Fig. 1:**
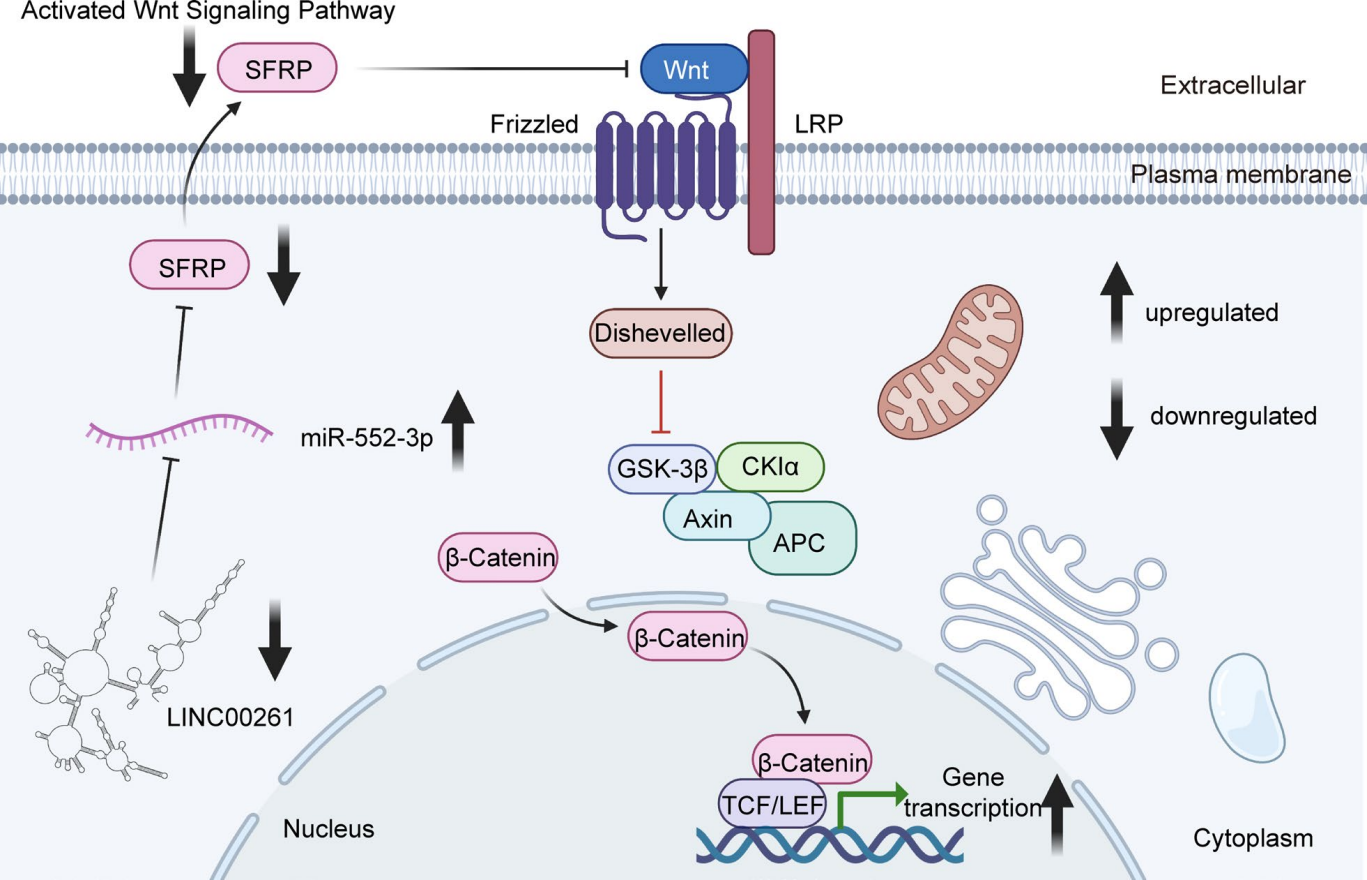
The downregulation of LINC00261 in non-small cell lung cancer accelerates tumor growth. LINC00261 sponges miR-522-3p, resulting in the modulation of SFRP expression. SFRP inhibits the activation of the Wnt signaling pathway, and accelerating apoptosis of NSCLC. The up arrows indicate upregulated and down arrows indicate downregulated

The overexpression of LINC00261 in PC was reported to significantly inhibit cell proliferation both in vitro and in vivo. Mechanistically, LINC00261 was found to bind to the bromo domain of p300/CREB-binding protein (CBP), preventing the recruitment of p300/CBP to promoter region of c-myc and decreasing the expression level of H3K27Ac. In this way, LINC00261 was responsible for reducing downstream c-myc transcription [47]. In addition, LINC00261 inhibition of cell proliferation and promotion of apoptosis were determined to be through sponging of miR-222-3p and reduction of c-myc expression [35]. Similarly, LINC00261 over-expression was shown to promote apoptosis by decreasing miR-23a-3p expression (a member of the mitogen-activated protein kinase [MAPK]-p38 signaling pathway) in PC cells [36]. In LC, the overexpression of LINC00261 was shown to overcome the inhibitory effect of miR-522-3p on corresponding mRNAs, and resulted in suppressed cell proliferation and accelerated apoptosis [34]. Mechanistically, LINC00261 has been shown to inhibit Wnt signaling (Fig. 1) [34], the miR-105/FHL1 axis [92], and the miR-1269a/FOXO1 axis [93]. As for Wnt signaling, LINC00261 participates it by promoting SFRP via ceRNA mechanism, and downregulated SFRP is proved to accelerate the tumorigenesis and metathesis of Choriocarcinoma [94]. SFRP inhibited the combination of Wnt ligand and Wnt receptor [95], and suppressed β-catenin expression. And when SFRP is inhibited, β-catenin expression level will recover [96]. In addition, inhibiting Wnt/β-catenin signaling pathway via other methods like Dickkopf-1 leads to similar effect on biological function compared with SFRP [97]. Thus, we consider that it is SFRP who mediated the degradation of β-catenin. Moreover, after being induced by forkhead box protein A2 (FOXA2), the overexpression of LINC00261 in lung adenocarcinoma (LUAD) cells slowed cell proliferation by inducing G2/M cell-cycle arrest [98].

Similarly, in BC, the reintroduction of LINC00261 was shown to arrest cell proliferation by protecting NME1 (a known tumor suppressor) mRNA from degradation [39]. Moreover, in HCC, Zhang et al. [79] showed that up-regulated LINC00261 significantly suppressed Notch signaling pathway by inhibiting Notch1 and Hes-1 expression. In endometrial carcinoma, LINC00261 was reported to inhibit cell proliferation by promoting the expression of forkhead box protein O1 (FOXO1) through a mechanism of reducing the levels of FOXO1-targeted miRNAs [37]. In addition, LINC00261 overexpression was also shown to promote apoptosis and decrease cell proliferation in choriocarcinoma [89]. In contrast to all other studies, one cholangiocarcinoma study reported that low LINC00261 expression actually increased cell apoptosis and inhibited cell proliferation [38]. Shahabi et al. [98] demonstrated that LINC00261 over-expression in LUAD cells slowed cell proliferation by inducing G2/M cell-cycle arrest. Interestingly, Chen et al. [81] speculated that low LINC00261 expression did not actually influence signaling pathways or gene expression related to cell proliferation and apoptosis, but that its high expression played a significant role in activating apoptosis and inhibiting cell proliferation. These ideas warrant further study.

### Cell motility

Cell motility refers to cancer-cell invasiveness into crucial organs. In general, LINC00261 expression may alter tumor-cell motility in many ways. The up-regulation of LINC00261 has been shown to increase cisplatin sensitivity in colon-cancer cells and to inhibit both cell invasion and cell migration [63]. LINC00261 expression has also been reported to sponge miR-550a-3p to regulate Serum deprivation response protein (SDPR), bringing about reduced invasiveness and migration of CD44^+^/CD24^−/low^ BC stem cells [40]. The overexpression of LINC00261 in endometrial carcinoma has been shown to inhibit both cell invasion and migration [37]. Moreover, down-regulated LINC00261 has been reported to significantly enhance cell migration and invasiveness in HCC cell lines [81]. The low expression of LINC00261 in both RBE and QBC939 cell lines also similarly suppressed cell invasiveness and cell migration [38]. When LINC00261 expression was reintroduced in NSCLC cells [34] and in choriocarcinoma cells [89], cell invasiveness was inhibited. Similarly, the overexpression of LINC00261 in LUAD cells [98], PC cells [47], and BC cells [39] also resulted in the inhibition of cell migration.

### Tumor angiogenesis

In prostate cancer, the overexpression of LINC00261 was shown to inhibit the transcription of dickkopf-related protein 3 (DKK3) by recruiting GATA binding protein 6 (GATA6), resulting in the inhibition of tumor angiogenesis; this action was reversed by silencing DKK3. Interestingly, in prostate cancer cells, DKK3 expression induced cellular quiescence through the activation of p38 MAPK signaling pathway [88]. These findings demonstrate that LINC00261 expression may suppress prostate cancer progression, and suggest that it may be a new biomarker for early diagnosis of prostate cancer.

### Chemoresistance

LINC00261 expression has been shown to increase the anti-tumor effect of cisplatin in colon cancer via the down-regulation of nuclear β-catenin. In addition, LINC00261 expression has been reported to inhibit the activation of Wnt signaling pathway, thereby promoting β-catenin degradation and blocking β-catenin translocation from the cytoplasm to the nucleus. Moreover, it is possible that the overexpression of LINC00261 may alleviate cisplatin resistance in colon-cancer cells by increasing the apoptosis rate [63]. Similarly, in human esophageal cancer, LINC00261 overexpression was observed to increase 5-FU drug sensitivity in tumor cells by modulating the methylation-dependent suppression of dihydropyrimidine dehydrogenase [87].

### Tumorigenesis

During both tumorigenesis and the EMT, LINC00261 has been shown to be co-regulated with FOXA2, so LINC00261/FOXA2 expression levels could potentially be used to predict both LUAD cell invasiveness and progression [99]. This study established the foundation for future research on the role of the LINC00261/FOXA2 axis in LC tumorigenesis by describing its capacity for tumor suppression. Moreover, in BC, only the down-regulation of LINC00261 expression was required to cause BC tumorigenesis. Mechanistically, LINC00261 may interact with NME1 (a known tumor suppressor) mRNA as protection against degradation, resulting in higher NME1 levels and increased tumor suppression [39].

### Molecular mechanisms underlying the functions of LINC00261

Many kinds of RNAs can bind to special miRNAs and regulate the gene expression, because they process miRNA recognition elements (MREs). These RNAs include lncRNAs, circRNAs, and pseudogenic RNAs, which are called competitive endogenous RNA (ceRNA) [100]. In this review, we found that LINC00261, acting as a ceRNA, sponge the miRNAs and downregulate the miRNAs level, further influencing the mRNAs level of target genes [101]. Many target genes of LINC00261 participate in multiple signaling pathway, such as Wnt/ β-catenin signaling pathway, p38 MAPK signaling pathway, and Notch signaling pathway. These signaling pathways regulated by LINC00261 are all related to tumor occurrence and progression.

### Wnt/β-catenin signaling pathway

Cooperating with other pathways, Wnt/β-catenin signaling pathway is highly conserved and plays a critical role in embryonic development and tumorigenesis of multiple cancers [102]. β-catenin is part of a complex of proteins that constitute adherens junctions (AJs). β-catenin also anchors the actin cytoskeleton and may be responsible for transmitting the contact inhibition signal that causes cells to stop dividing once the epithelial sheet is complete. Therefore, when Wnt/β-catenin signaling pathway is dysregulated, cell proliferation will be no longer restrained even if the epithelial sheet is complete [103]. Wang et al. [63] indicated that LINC00261 could inactivate Wnt pathway through regulating β-catenin at the transcriptional level. They also found LINC00261 can promote degradation of β-catenin and inhibit it in nuclei. Moreover, LINC00261 could recruit GATA6 and suppress DKK3 expression. And DKK3 can interact with miRNA and regulate Wnt/β-catenin signaling pathway [88]. Shi et al. [34] also revealed that LINC00261 bound to miR-522‐3p and inhibited Wnt signaling pathway, alleviating progression of NSCLC cells.

### Notch signaling pathway

Notch signaling pathway can directly connect events which happen on the cell membrane and transcriptional regulation. And the function of Notch signaling in different environments is based on the ability to influence the development and selection between adjacent cells [104]. Notch signaling pathway is related to tumor cell proliferation, differentiation, and metastasis. NOTCH was also conversed in multiple mammals, especially in mice, encoding a series of cell membrane receptors. Zhang et al. [79] recently demonstrated that upregulating LINC00261 prominently inhibited Notch1 and Hes-1 expression in HCC cells, which are vital members of Notch signaling pathway. And Hes-1 is a key downstream target of Notch signaling pathway and can be activated by the release and transport of the Notch intercellular domain, which interacts with the transcriptional complex [105, 106]. Therefore, increased expression of LINC00261 suppressed Notch signaling pathway to exert tumor suppression function in HCC.

### P38 MAPK signaling pathway

P38 MAPK signaling pathway plays a critical role in the regulation of many cellular functions, such as cell growth, differentiation, and respond to stress and inflammatory reaction. For prostate cancer, Li et al. [88] indicated that LINC00261 promoted DKK3 transcription expression by recruiting GATA6, and DKK3 could induce cellular quiescence and inhibit tumor progression through activating the p38 MAPK signaling pathway. DKK3 promoted p-p38 nuclear translocation [107] and mediated the activation of Wnt/β-catenin signaling pathway, which is closely interacted with many other signaling pathways, causing cell proliferation, migration and invasion of PCa [108].

## Conclusions

**Fig. 2:**
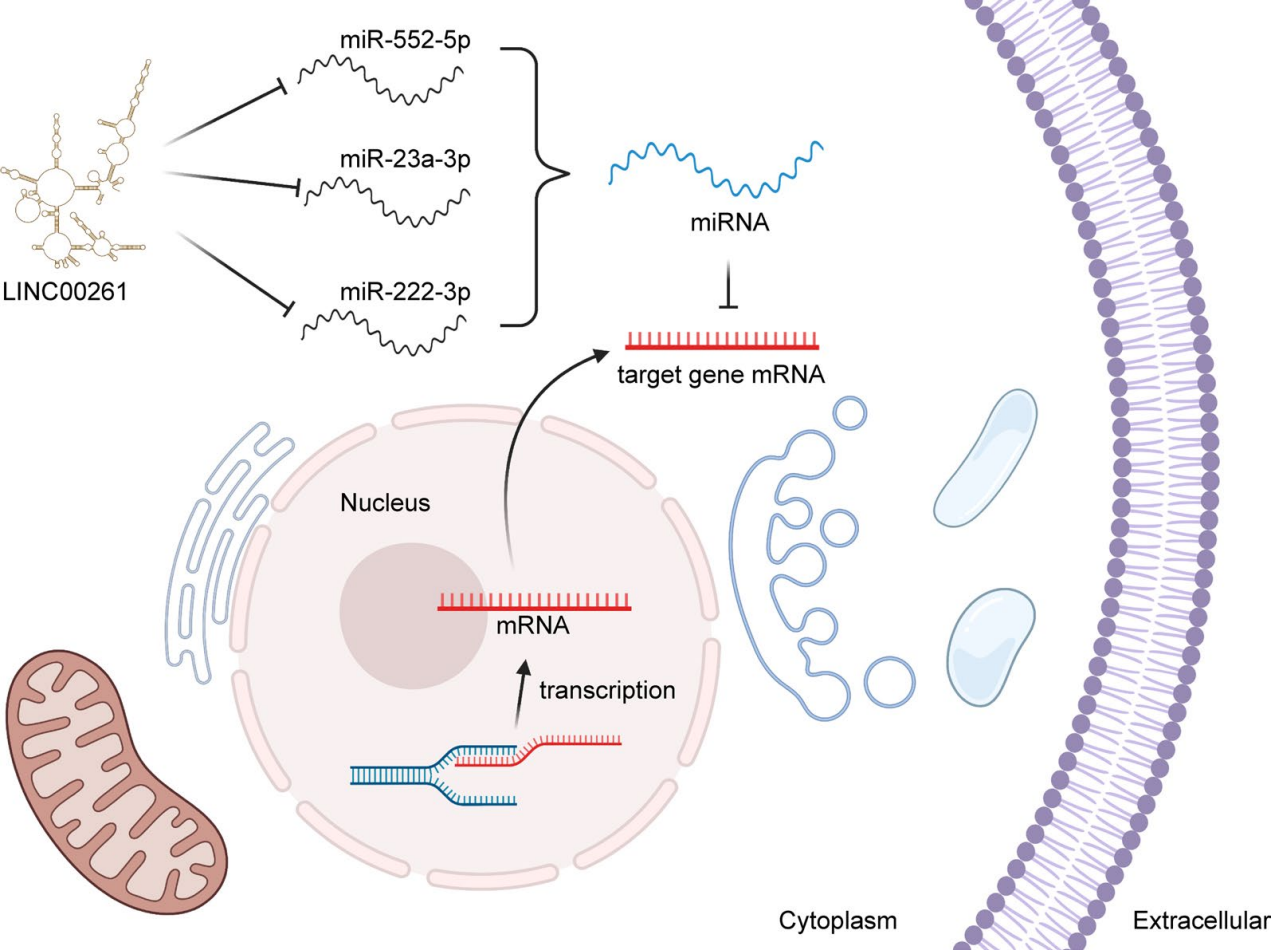
The molecular-mechanism map of LINC00261 in pancreatic cancer. LINC00261 binds to miR-552-5p, miR-23a-3p, and miR-222-3p separately to promote the expression of tumor-suppressor genes, including FOXO3, FOXA2, E2F, and IGF2BP1 to inhibit cancer progression

Considerable researches have shown that lncRNAs play vital roles in both tumorigenesis and tumor development via the regulation of gene expression [109–111]. In view of the fact that LINC00261 can be promoted by the demethylation of promoter region, we summarized the roles of LINC00261, a tumor suppressor gaining increasingly important status, in cancer research. Although LINC00261 was first identified approximately eight years ago [112], its roles as a novel lncRNA involved in a variety of diseases and its molecular mechanisms have only been reported recently. The present LINC00261 review indicates that its expression is widely low expressed in many cancer types (e.g., PC, GC, LC, CRC, BC, and HCC). At the same time, LINC00261 expression has been found to be correlated with clinicopathological characteristics (e.g., tumor stage, lymph-node metastasis, patient survival, and tumor prognosis). As a tumor suppressor, LINC00261 contributes to modulating cancer-cell biology via multiple molecular mechanisms (e.g., cell proliferation, apoptosis, invasiveness, migration, chemoresistance, and tumorigenesis). Mechanistically, the sponging and binding to miRNAs indicates that LINC00261 acts as a ceRNA to inhibit the expression of cancer-related genes (Fig. 2). The evidence that LINC00261 plays an antineoplastic role in a variety of human cancers indicates that it has the potential to be a target for both cancer diagnosis and treatment and could become a biomarker for many types of cancer.

## Data Availability

All data are included in the article.

## Abbreviations

lncRNAs: Long noncoding RNAs
LINC00261: Long intergenic non-protein coding RNA 261
ncRNAs: Noncoding RNAs
miRNAs: microRNAs
circRNAs: Circular RNAs
mRNA: Messenger RNA
EMT: Epithelial-mesenchymal transition
PC: Pancreatic cancer
GC: Gastric cancer
CRC: Colorectal cancer
LC: Lung cancer
NSCLC: Non-small-cell lung cancer
HCC: Hepatocellular carcinoma
BC: Breast cancer
5-FU: 5-fluorouracil
CBP: CREB-binding protein
FOXA2: Forkhead box protein A2
LUAD: Lung adenocarcinoma
FOXO1: Forkhead box protein O1
SDPR: Serum deprivation response protein
DKK3: Dickkopf-related protein 3
GATA6: GATA binding protein 6
MREs: miRNA recognition elements
ceRNA: Competitive endogenous RNA
AJs: Adherens junctions
